# Potential for Improving Potency and Specificity of Reovirus Oncolysis with Next-Generation Reovirus Variants

**DOI:** 10.3390/v7122936

**Published:** 2015-12-01

**Authors:** Adil Mohamed, Randal N. Johnston, Maya Shmulevitz

**Affiliations:** 1 Department of Medical Microbiology and Immunology, University of Alberta, Edmonton, AB T6G 2E1, Canada; adilm@ualberta.ca; 2 Department of Biochemistry and Molecular Biology, Cumming School of Medicine, University of Calgary, Calgary, AB T2N 4N1, Canada; rnjohnst@ucalgary.ca

**Keywords:** reovirus, oncolytic virus, cancer, attachment, uncoating, replication, reverse genetics

## Abstract

Viruses that specifically replicate in tumor over normal cells offer promising cancer therapies. Oncolytic viruses (OV) not only kill the tumor cells directly; they also promote anti-tumor immunotherapeutic responses. Other major advantages of OVs are that they dose-escalate in tumors and can be genetically engineered to enhance potency and specificity. Unmodified wild type reovirus is a propitious OV currently in phase I–III clinical trials. This review summarizes modifications to reovirus that may improve potency and/or specificity during oncolysis. Classical genetics approaches have revealed reovirus variants with improved adaptation towards tumors or with enhanced ability to establish specific steps of virus replication and cell killing among transformed cells. The recent emergence of a reverse genetics system for reovirus has provided novel strategies to fine-tune reovirus proteins or introduce exogenous genes that could promote oncolytic activity. Over the next decade, these findings are likely to generate better-optimized second-generation reovirus vectors and improve the efficacy of oncolytic reotherapy.

## 1. Introduction

### 1.1. Reovirus Naturally Infects the Small Intestine

Mammalian orthoreovirus (MRV, herein referred to as reovirus) is a non-enveloped, icosahedral virus in the *Reoviridae* family. Reovirus poses little-to-no threat as a human pathogen, but has been used as a model system to improve our understanding of viruses and cells. The first Respiratory Enteric Orphan (REO) virus was isolated from healthy children in the 1950s [1,2]. Three prototypic serotypes of reovirus have since been well characterized: serotype 1 Lang (T1L), serotype 2 Jones (T2J), and serotype 3 Dearing (T3D). Reovirus is ubiquitous, with field isolates readily found in water bodies around the world, in addition to human, bovine, porcine, and murine fecal samples [3,4,5,6,7,8]. Reovirus genomes are composed of 10 dsRNA segments, and consequently natural isolates of reovirus tend to be genome reassortants. Humans are exposed to reovirus during childhood, resulting in up to 100% seropositivity among adults [9,10,11]. While other members of the *Reoviridae* family, such as orbiviruses and rotaviruses, cause gastrointestinal disease in livestock and humans respectively, reovirus has not been associated with disease during natural infection of humans or animals.

Reovirus has evolved some remarkable strategies to survive in, and even exploit the unique and harsh conditions of the intestine. Intestinal epithelial cells are polarized, with specialized apical membranes coated by a thick glycoprotein layer that conceals surface receptors and creates a barrier to viruses. Reovirus therefore exploits the antigen-transporting activity of specialized microfold epithelial (M-) cells to gain access to the basolateral membranes of intestinal epithelial cells [12,13,14,15,16,17]. Both M-cells and enterocytes stain positive by immunohistochemistry in reovirus-fed mice. Trimeric σ1 cell attachment proteins protrude from reovirus vertices, and facilitates binding to α2-3-linked sialic acid on M-cells [13] and to Junction Associated Molecules (JAM-A) [18] on basolateral epithelial membranes.

**Figure 1:**
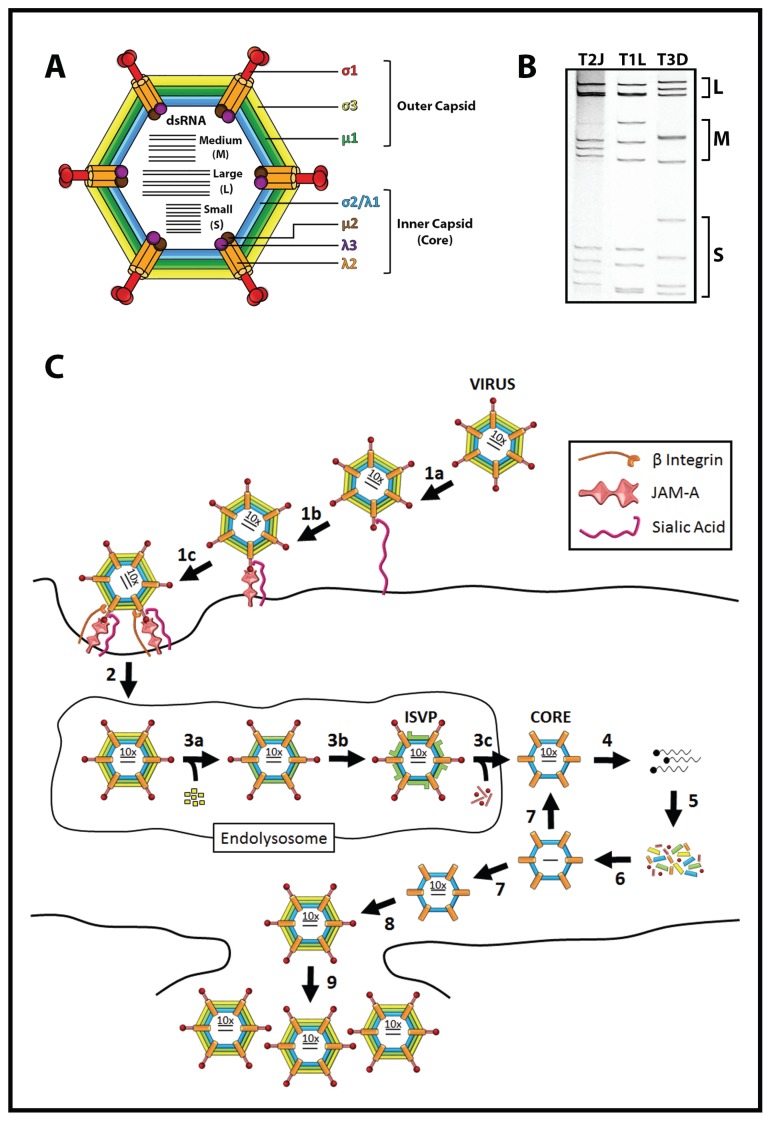
Reovirus structure and replication. (**A**) Reovirus outer capsid and inner capsid proteins; (**B**) Genomic dsRNA profiles of prototypic reovirus serotypes 1 Lang, 2 Jones, and 3 Dearing; (**C**) Life cycle of reovirus. Cell attachment is mediated through (1a) sialic acid binding, (1b) JAM-A association, and (1c) integrin binding; (2) Reovirus is internalized through endocytosis. Within late endosomes/lysosomes; (3) uncoating proceeds through (3a) proteolytic-mediated removal of σ3, (3b) cleavage of µ1 to δ, and (3c) loss of σ1 concomitant with membrane penetration and transition to cores; (4) Viral +sense RNAs are transcribed and released by cytoplasmic core particles; (5) Reovirus proteins are expressed and (6) assemble with +sense RNAs into progeny core particles; (7) Genomic dsRNA is synthesized within progeny cores, which serve to amplify reovirus macromolecular synthesis; (8) Reovirus is fully assembled through addition of outer-capsid proteins and released from cells by lysis and/or apoptosis.

Reovirus has a very complex capsid shell composed of 6 structural proteins (σ1, σ2, σ3, µ1, λ1, λ2) that interlock into two concentric layers; the inner- and outer-capsids (Figure 1). The RNA-dependent RNA polymerase (λ3) and the multifunctional µ2 protein are packaged inside the virion at each of 12 vertices. The complexity of reovirus capsids likely contributes to stability under harsh environmental and enteric conditions. Reovirus enteric infection involves proteolytic processing of the virion by enzymes in the intestinal lumen. Chymotrypsin and trypsin completely degrade the outer-most protein σ3 and cleave the underlying µ1 protein into membrane-penetrating fragments. The resulting intermediate virions, called infectious subviral particles (ISVPs), can penetrate both surface- and endocytic-membranes. The observation that freshly made ISVPs are more infectious than whole virions supports the evolutionary compatibility of reovirus towards the enteric niche [2,19]. Unlike other virus families, reovirus does not fully disassemble. Instead, ISVPs convert into core particles composed of the inner-capsid proteins and dsRNA genome. The core proteins serve to conceal genomic viral dsRNA from the host, which would otherwise stimulate a rapid and potent antiviral response. Reovirus core proteins (polymerase λ3, capping enzyme λ2 [20], and co-factors µ2 [21] and λ1 [22]) then transcribe and release viral capped mRNAs into the cytoplasm through channels created by pentameric λ2 proteins at each vertex. Reovirus proteins are expressed by the host translation machinery, and assemble into progeny cores that further amplify replication (Figure 1). Ultimately, outer capsid proteins are assembled onto cores to produce complete virions, which are released into the intestinal lumen for transmission. The ability of reovirus to bind multiple receptors on unique intestinal cells and to use intestinal enzymes for defined disassembly provide noteworthy examples of how reovirus is intimately adapted for intestinal infections.

### 1.2. Reovirus Is a Promising Cancer Therapy

The use of viruses to treat cancer was suggested a century ago when cancer patients occasionally went into remission following natural virus infection or vaccination [23]. Several viruses including reovirus, vaccinia virus, Newcastle disease virus, adenovirus, Maraba virus and vesicular stomatitis virus demonstrate tumor-specific cytolysis and are therefore candidate cancer therapies. Most oncolytic viruses show specificity towards cancer cells either because they are non-human-tropic, or because they are attenuated or genetically modified. Interestingly, reovirus naturally infects humans yet is inherently more infectious towards transformed over normal cells [24,25].

Normal mouse fibroblasts become permissive to reovirus infection when first transformed by constitutively active H-Ras [26,27], and provide a well-controlled model system for discovering the molecular basis of reovirus specificity towards transformed cells. Over 18 pathways downstream of Ras promote cell growth by modulating a compendium of cellular processes [28]. A comparison of each step of reovirus replication among Ras- *vs.* non-transformed cells revealed that (1) lysosomal proteolysis of incoming reovirions is enhanced by Ras-transformation [29,30]; (2) progeny virus particles produced in Ras-transformed cells are more infectious than progeny produced in non-transformed cells [29]; (3) Ras-transformed cells are more susceptible to reovirus-induced apoptosis [29,31]; and (4) Ras-transformed cells exhibit impaired IFN expression, thereby permitting efficient cell-to-cell spread of reovirus [32,33]. These studies reveal that cellular changes induced by activated Ras signaling create *multiple* Achilles heels that are ultimately exploited by reovirus for efficient replication. The existence of multiple barriers helps ensure that reovirus replication is strongly tumor-specific. With regards to engineering improved reovirus variants, one should ensure that these four mechanisms of specificity are not jeopardized.

Wild type reovirus demonstrated promising anti-tumor activity in a large assortment of pre-clinical mouse tumor models [34,35,36,37,38]. A Canadian-based biotechnology company, Oncolytics Biotech Inc. (Calgary, AB, Canada), has initiated human clinical trials using wild-type reovirus under the trade-name Reolysin^®^. Twenty-six phase I/II clinical trials have since been conducted, some ongoing, in an assortment of cancers. Reovirus was found to be well tolerated following both intratumoral and intravenous administration, and show preliminary evidence of anti-tumor activity in patients [39,40,41,42,43,44,45,46]. A phase III clinical trial in head and neck squamous cell carcinoma was recently completed [47].

### 1.3. Optimizing Reovirus Oncolysis

Despite promising suggestions of antitumor activity, response to reovirus monotherapy in mouse models of aggressive cancers and in human trials of advanced malignancies are described as modest and short-lived [39,40,41,42,43,44,45,46]. On one hand, it is not surprising that phase I and II trials show incomplete response, given the severity of disease among patients in such trials. Conversely, these findings support that advancements to reovirus oncolysis might improve efficacy. Important to this review, all trials currently use the P.W.Lee laboratory strain of reovirus serotype 3 Dearing (T3D) as the wild-type form (T3wt). T3wt provides a practical strategy for proof-of-concept and to obtain FDA approval, as wild-type reovirus is well characterized as non-pathogenic and provides some clinical benefit when used in combination therapy. However, considering that reovirus is exquisitely well adapted for its natural gastrointestinal niche, it seems logical that modifications to reovirus could promote adaptation and potency in the new human-imposed therapeutic cancer niche. Accordingly, development of “next-generation” reovirus variants that exhibit enhanced replication in tumor cells, especially in combination with complementary strategies that reduce immune-clearance and boost anti-tumor immunity, are likely to promote the success of reovirus oncotherapy.

Many studies have demonstrated the benefit of combining reovirus with conventional therapies such as radiation and chemotherapy [41,42,48,49,50]. Checkpoint blockade is a promising immunotherapeutic strategy that enhances anti-tumor immunity and was recently observed to strikingly improve reovirus efficacy in a pre-clinical mouse tumor model [51]. A long-standing belief by researchers is that combination of multiple cancer-targeting viruses, likely in tandem, would help overcome immune-clearance of individual viruses and harness the combined anti-tumor activities of each virus. This review strives to provide a comprehensive analysis of strategies to modify reovirus itself for improved oncolytic potency and safety. Ultimately, optimal viral oncotherapy will likely incorporate both changes to the virus and combination with other therapeutic approaches.

## 2. Cell Attachment

**Figure 2:**
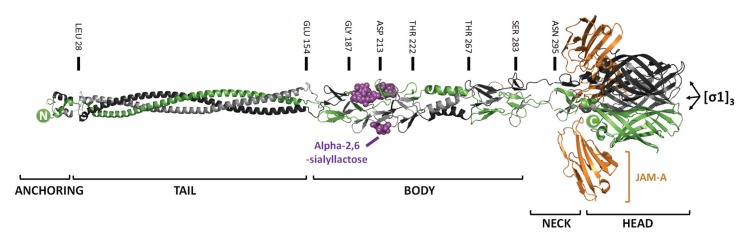
Structure and function of distinct domains in reovirus σ1 trimers. Trimers of σ1, with monomers colored green, grey, and black. Positions of amino acids that define distinct structural domains are provided. Predicted structure of the N-terminal virion-anchoring domain (residues 1–27) and coiled-coil σ1 (residues 27–154) are depicted (coiled-coil crystal structures from the unrelated trimeric autotransporter adhesins, PDB 2YO3, were used for depiction) [52]. The body domain of T3D σ1 (residues 154–283) bound to α-2-6-sialyllactose (purple) and the neck domain (residues 283–295) are depicted from crystal structure PDB 3S6Y [53]. The C-terminal head domain in complex with JAM-A (orange) are from PDB 3E0Y [54]. N- and C-termini are indicated by green circles.

Attachment of reovirus to cells is mediated through at least three distinct interactions between virus and host cells. Trimeric σ1 cell attachment proteins protrude from reovirus particle vertices, held in place by interactions between the N-terminal anchoring domain of σ1 and the pentameric λ2 channel proteins. Intrinsic oligomerization of σ1 is mediated through a coiled-coil heptad repeat domain referred to as the reovirus tail domain (Figure 2) [55,56]. The body and neck domains, composed of β-sheet-α-helix-β-sheet structures, are responsible for binding to sialic acid [57]. The neck domain is also subject to proteolysis in some isolates of T3D [58]. The globular head domain mediates interactions with the cellular tight junction associated protein, junction adhesion molecule-A (JAM-A) [18,59,60]. Recently it was found that σ1 head can also bind to Nogo receptor NgR1on neuronal cells [61]. Finally, the extra-virion domain of λ2 binds β1 integrins to permit firm adhesion and initiate endocytosis-mediated uptake into cells [62,63].

### 2.1. Mutations that Modulate Sialic Acid Binding

Binding of σ1 to sialic acids is thought to mediate initial reovirus adhesion to target cells [57,64]. The body domain of reovirus T3D binds a range of sialylated glycans such as sialoglycophorin and sialosides with terminal N-acetylneuroaminic acid (NeuNAc) linked in either an α-2,3 or α-2,6 configuration [57,65,66,67]. Specifically, amino acids at positions 198, 202 and 204 are implicated in binding of T3D σ1 to sialic acids, since mutation of these residues (N198D, R202W, and P204L) eliminates reovirus-sialic acid interactions [68]. In contrast, T1L binds exclusively to GM2 sialylated glycans, and this binding is mediated through the σ1 neck domain [53,65,69,70].

To assess the contribution of sialic acid binding for reovirus replication *in vitro* and *in vivo*, Dr. Terence Dermody’s laboratory (Vanderbilt University) made use of a serotype 3 field isolate (T3C44, also called T3SA^−^) that is deficient in sialic acid binding. Using serial passage in MEL cells, which only support replication of sialic acid-binding reovirus strains, the laboratory isolated an isogenic T3C44 variant (T3C44-MA, also called T3SA^+^) that gained sialic acid binding through a L204P mutation in σ1 (Table 1) [57]. Similarly, sialic acid-binding (SA^+^) *vs.* non-binding (SA^−^) isogenic pairs were created by introducing an R202W point mutation into wild-type serotype 3 Dearing strain (T3D). Both T3SA^+^ and T3SA^−^ replicated equally well on L cells, which support sialic-acid independent entry, but T3SA^+^ replicated considerably better in HeLa cells relative to T3SA^−^. T3SA^+^ and T3SA^−^ variants were then compared in a neonatal immunocompromised mouse model, where reovirus infection can spread to the brain, heart and liver and exhibit pathogenesis. T3SA^−^ showed altered cell tropism, decreased virus dissemination, and reduced pathogenesis relative to T3SA^+^ [61,71,72]. These studies demonstrate that loss of sialic acid binding can negatively impact reovirus infectivity, though the impact in various tumor models remains to be tested.

Sialic acid binding most likely also plays an important role during reovirus infection of tumor cells, though this has yet to be tested directly. Hypersialylation and incorporation of dietary non-human sialic acid Neu5Gc are common among tumor cells and implicated in tumor growth and immune escape [73,74]. Hypothetically, improving the strength of sialic acid binding or sialylated glycan specificity, for example by modifying the T3 sialic acid binding domain or combining the distinct SA binding domains of T1 and T3 serotypes, could further promote specificity and potency of reovirus oncolysis.

Through persistent infection of Vero monkey kidney cells with T3D, Sandekian and Lemay (2015) isolated a reovirus variant (VeroAV) that may provide advantage at the sialic acid binding stage of reovirus infection. VeroAV contains unique mutations in the coiled-coil tail (Q78P) and sialic acid binding (N198K) domains of σ1 [75,76]. Interestingly, these mutations in σ1 were found to improve reovirus infectivity towards Vero cells, especially when combined with co-adapted mutations in the µ1 outer capsid protein. Binding of VeroAV to Vero cells was stronger than wild-type reovirus and was sensitive to neuraminidase treatment. In the future, hemagglutination assays, surface plasmon resonance, or glycan arrays could be used to directly examine if the N198K mutation promotes enhanced sialic acid binding strength and/or specificity. If sialic acid binding is found to be superior for VeroAV, it would be interesting to determine if this variant replicates better than wild-type reovirus on HeLa and MEL cells (which poorly support T3SA^−^ viruses) and especially, on a panel of cancer cells that differ in availability of attachment factors (sialic acids, JAM-A, and integrins).

**Table 1:** Reovirus variants with altered infectivity.

	Step	Variant Name	Mutations (Amino Acid Change) ^1^	Domain Function	Phenotype
**Improving Potency**	**Attachment**	T3DSA^−^	σ1 (R202W)	Sialic-binding	Reduced infection in some cells (e.g., HeLa) and *in vivo* pathogenesis in neonatal immunocompromised mice
		VeroAV	σ1 (Q78P; N198K)	Trimerization; Sialic-binding	Enhanced binding to Vero Cells
			µ1 (E89G; A114V)		
		Jin-1	σ1 (T193M; Q336R)	Sialic-binding; JAM-A binding	Infectious towards JAM-A deficient cells
		Jin-2	σ1 (G187R; Q336R)	Sialic-binding; JAM-A binding	
		Jin-3	σ1 (G196R)	Sialic-binding	
		T3D-S1His	σ1 ((His)_6_ tag @ C-terminus)	Additional binding domain added	Ability to replicate in JAM-A-deficient U118 cells that express (His)_6_-specific antibody fragment
	**Uncoating and Onset of Infection**	NA	µ1 (A305L), (A276V), (D371N), (Q456R), (P497S), (L185S), or (E89Q)	µ1-µ1 interactions	Altered rates of ISVP → ISVP* and core production
		Y354H	σ3 (Y354H)	C-terminal surface exposed domain	Enhanced disassembly and resistance to E64 protease inhibitor. Enhanced replication, dissemination and pathogenesis in immunocompromised mice
		T3v1	λ1 (N138D)	Inner face of virion core	Enhanced particle infectivity and oncolytic activity *in vivo*
			λ2 (M1101I)	Flap domain that open/close	
			λ3 (P400S)	Core-facing surface	
		T3v2	σ1 (S18I)	Virion-anchoring domain	
**Improving Specificity/Safety**	**Attachment**	HTR1 (AV-Reo)	σ1 (L116P; V127A; Q251STOP; I300M) σ3 (S177F; H251L)	Trimerization; JAM-A binding	Reduced toxicity *in vivo*
	**Antiviral Response**	P4L-12	σ3 (G198E; M221I)		Increased IFN-sensitivity. Improved specificity towards IFN-deficient Ras-transformed cells
			µ1 (P315S; T449A)		
			µNS (V705A)		
			λ2 (T636M)	Methyltransferase domain	
		NA	µ2 (P208)	Unknown	Important in repression of interferon signaling
		NA	σ3 (R236), (R239), (K291), or (K293)	dsRNA binding domain	
		ts453	σ3 (N16K)	µ1 association domain	Increased dsRNA binding and IFN resistance

^1^ Mutated residues in bold underline are suggested to play a dominant role in the variant phenotype.

### 2.2. Modifications that Alter Binding to JAM-A or Promote JAM-A Independent Attachment

Following low-affinity reovirus σ1 adhesion to sialic acids, high-affinity binding is attained by binding of the globular σ1 head domain to JAM-A (also called JAM-1 or F11). JAM-A is a cell adhesion molecule with diverse functions such as tight junction formation, leukocyte migration and platelet activation [77].

Many studies have found that JAM-A expression is common in cancers and contributes to proliferation, metastasis, and poor prognosis [78,79,80,81,82,83,84]. For example, 82% of cancers covering a wide range of origins stain positive for JAM-A expression according to the Human Protein Atlas (HPA) [85,86,87,88,89]. Terasawa *et al.* found that among 19 tumor cell lines, 18 were positive for JAM-A expression and that JAM-A did not correlate with differential susceptibility of these cells to reovirus-induced cell death [90]. However, some cancers do show limited accessibility to JAM-A, which would pose a restriction to reovirus oncolysis. For example, van Houdt *et al.* (2008) found that patient-derived colorectal and liver metastases have cytoplasmic retention of JAM-A using tissue microarrays [91]. Among the cancers evaluated in the HPA, gliomas and melanomas seemed to express relatively limited JAM-A. Accordingly, reovirus variants that can attach to cells in a JAM-A-independent manner could provide improved therapies towards JAM-A-deficient cancers.

Using forward genetics, van den Wollenberg *et al.* (2012) isolated three reovirus variants that can replicate on JAM-A negative U118 human glioblastoma cells [92]. All three JAM-A independent (jin) variants had mutations proximal to the sialic acid binding motif of the σ1 cell attachment protein (T193M, G187R, and G196R in jin-1, jin-2, and jin-3 respectively, Table 1). Variants jin-1 and jin-2 also shared a common secondary mutation, Q336R, in the JAM-A binding domain of σ1. Jin-1 was capable of infecting a panel of cancer cells that resist wild-type reovirus infection. Conversely, plaques formed by jin-1 and jin-2 mutants were actually smaller than T3wt on JAM-A expressing human embryonic retinoblast 911 cell line, suggesting that benefits of jin-associated mutations are cell-specific. It would be interesting to evaluate expression of JAM-A and other junction adhesion molecules on the cell panel used in this study, and determine if a reciprocal correlation exists between JAM-A expression and fitness of jin-over wild-type reovirus. Importantly, the jin mutants did not replicate in primary human fibroblasts, demonstrating retained specificity towards tumor cells. Infection by jin mutants was sialic-acid dependent, as treatment with wheat germ agglutinin to occlude sialic acid binding decreased infectivity. It will be necessary to perform attachment assays to directly implicate increased cell binding as the advantage for jin mutants, as these mutations could provide un-anticipated benefits for other stages of reovirus entry and infection. If binding is the basis for enhanced infectivity, it is possible that jin mutants use an auxiliary receptor, that the modifications increase sialic acid affinity, or that the changes reduce virion-cell distance to permit enhanced binding between λ2 and integrins.

An alternative strategy to facilitate high-avidity binding of reovirus to JAM-A-deficient cancer cells would be to introduce extrinsic receptor binding domains into σ1. The strategy of re-engineering virus cell-attachment proteins (transductional targeting) has worked well for oncolytic adenovirus to overcome limited expression of its receptor, coxsackievirus and adenovirus receptor (CAR), on tumor cells. In fact, since the adenovirus fiber and reovirus σ1 cell-attachment proteins show structural similarity, the JAM-A binding head domain of reovirus was successfully fused to the adenovirus fiber tail to re-target adenovirus [93,94,95,96,97]. As a proof-of-concept for feasibility of transductional re-targeting in reovirus, van den Wollenberg (2008) added a hexahistidine (His) tag to the C-terminus of σ1 [98], and then expressed a single-chain His-tag-specific antibody fragment on the surface of JAM-A-deficient U118 glioblastoma cells. Strikingly, U118 cells became permissive to reovirus when alternative adhesion mechanisms were provided. As discussed in a section that follows, the advent of an efficient reverse genetics system for reovirus will likely permit additional ingenuity with respect to transductional re-targeting for JAM-A deficient cancers.

## 3. Virus Disassembly, Membrane Penetration, and Establishment of Infection

### 3.1. Reovirus Uncoating Contributes to Specificity and Potency of Oncolysis

Within minutes of oral ingestion, reovirus exploits the intestinal proteases trypsin and chymotrypsin to cleave outer capsid proteins and undergo structural changes that ultimately permit membrane penetration, production of transcriptionally active core particles, and onset of viral macromolecular synthesis (Figure 1) [99,100]. The first intermediates produced during reovirus uncoating are infectious subvirion particles (ISVPs), and requires the complete degradation of outer-most capsid protein σ3 and cleavage of the under-lying µ1 protein [101]. In ISVPs, the myristoylated µ1N fragment (residues 1–2), the δ fragment (residues 43–581) and the short C-terminal ϕ fragment remain particle-associated. Host membrane exposure promotes formation of ISVP* through conformational changes in µ1 and release of membrane-pore-forming µ1N and ϕ fragments from virus particles [102,103,104,105,106,107,108,109,110,111]. The importance of releasing the myristoylated µ1N fragment and ISVP* formation is demonstrated by the dramatic loss of infectivity by reovirus particles with mutations that prevent µ1 processing [104,107]. Finally, membrane penetration liberates remaining outer-capsid proteins δ and σ1 from virions [107], concomitant with opening of the λ2 channels at each vertex through which newly synthesized viral RNAs are extruded into the cell cytoplasm to establish infection. The complex steps of reovirus uncoating have been extensively studied and are reviewed thoroughly elsewhere [112,113].

In the absence of intestinal proteases, such as during infection of cells in culture, disassembly of reovirus requires endocytosis and proteolysis by lysosomal cysteine proteases cathepsin B and L [90,114,115]. In mice models where reovirus causes pathogenesis, knock-out of cathepsins or use of cathepsin inhibitors affected viral spread among organs and mouse survival [116], indicating that non-intestinal proteases can affect reovirus infectivity *in vivo*. Furthermore, proteases such as airway trypsin-like protease (HAT) on respiratory cells, neutrophil elastase, cathepsin S, neutrophil cathepsin G, and mast cell chymase available during inflammation by infiltrating immune cells can facilitate reovirus uncoating and might contribute to ISVP formation in their respective environments [117,118,119]. However, intestinal proteases have unique cleavage site preferences and are presumably very abundant [120,121,122], so it is currently unclear whether reovirus disassembly in lysosomes *versus* extracellularly by non-intestinal proteases are equally efficient especially *in vivo*. For example, Nygaard *et al.* (2011) found that despite ISVP production by HAT *in vitro*, progeny titers from lungs of mice intranasally infected by complete reovirions were significantly lower than titers from ISVP-infected mice, suggesting inefficient virus uncoating in the murine respiratory tract [119]. Interestingly, pre-treatment of mice with lipopolysaccharide or UV-inactivated reovirus to induce inflammation and immune cell-associated proteases increased conversion to ISVPs in bronchoalveolar lavage fluid, assessed by the proportion of viruses that became resistant to E64 broad-spectrum cysteine protease inhibitor. Progeny virus titers were also significantly increased, and correlated with increased numbers of monocytes. These results indicate that the uncoating efficiency of reovirus is dependent not only on the identity and abundance of proteases available at the site of virus replication, but also by proteases introduced by immune cells.

While reovirus uncoating is one of the most-studied steps of reovirus replication, much less is known about the efficiency of this stage within the tumor environment. Early studies into the molecular basis for reovirus specificity towards transformed cells found that addition of RasV12 oncogene by itself was sufficient to increase cleavage of µ1 to δ and onset of reovirus infection by 3–4-fold [29]. Furthermore, cancer cells that were less susceptible to reovirus infection, such as U87 and U118 glioma cells, became infected when exposed to ISVPs generated by *in vitro* proteolysis [30,123]. Similarly, tissue fragments and single-cell populations of patient-derived colorectal tumor cells were susceptible to reovirus ISVPs but not whole virions [91]. A comparison of 19 human cancer cell lines found that susceptibility to reovirus infection and cytopathic effect correlated with active levels of cathepsins B and L [90]. Together, these studies demonstrate that efficiency of uncoating contributes to reovirus specificity towards tumor cells over non-transformed cells, and that the availability of proteases in cancer cells further modulates the extent of reovirus infectivity. 

With respect to tumors *in vivo*, many extracellular proteases have been found to increase in expression and extracellular distribution. However, it is currently unclear whether extracellular and intracellular proteases produced by tumor and tumor-associated immune cells are of sufficient type and abundance to efficiently uncoat reovirus. Intriguingly, while U118 glioma cells could not support infection by whole virions in cell culture, *in vivo* subcutaneous tumors derived from U118 cells effectively regressed by a single intratumoral dose of reovirions [30]. Cathepsin B activity in both U118 and U87 glioma cells was notably higher in implanted tumors than cells grown in culture. An independent study found that spheroids derived from U118 cells also became susceptible to reovirus infection and had increased levels of cathepsins [124]. These studies suggest that the tumor microenvironment might support reovirus uncoating even when cells in culture cannot. Further studies are needed among distinct tumors, to fully appreciate the extent to which tumor proteases are sufficient or insufficient for maximal reovirus infectivity, dissemination, and oncolysis.

### 3.2. Mutations in σ3 Promote Reovirus Uncoating

**Figure 3:**
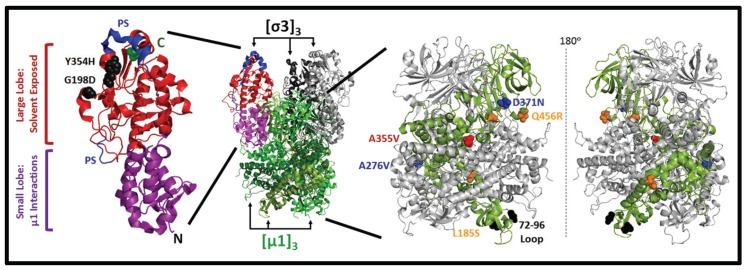
Structural and functional domains of reovirus σ3 and µ1 outer capsid proteins. (**Middle**) Crystal structure of µ1 timers (light, medium and dark green) in association with σ3 (black, grey, and multi-color) in T1L reovirus (PDB pdb1jmu) [126]; (**Left**) Domains of σ3 from T3D reovirus (PDB 1FN9) are depicted including two protease hypersensitive regions (PS, blue) [128]. Positions of two mutations G198D and Y354H discussed in the text are indicated with black spheres. N- and C-terminus (green sphere) are indicated; (**Right**) Associations between three µ1 monomers (one is green, two others are grey) are depicted in mirror-images. The 72–96 loop (black), and mutations that lie between µ1 trimers (blue) or between µ1 monomers (orange), discussed in the text, are highlighted with spheres.

Reovirus variants with distinct abilities to accomplish specific steps of uncoating could provide important information on the efficiency of uncoating in tumors, and perhaps provide a tool to promote the potency of reovirus oncolysis. Many distinct mutations have been found to affect reovirus uncoating, though to our knowledge, these reovirus variants have yet to be evaluated relative to wild-type reovirus in tumor models. The outermost outer-capsid protein, σ3, is thought to protect the underlying µ1 protein from prematurely undergoing conformational changes needed for membrane penetration [125]. The initial step of reovirus uncoating therefore requires the degradation of σ3. Many studies have revealed the importance of distinct structural domains of σ3 [108,126,127,128,129]. Sigma 3 is composed of a small lobe (Figure 3, resides 1–90 and 287–336, purple) that coordinates zinc binding (resides 51, 54, 71, and 73) and interacts with µ1. The larger lobe (Figure 3, residues 91–286 and 337–365, red) protrudes from the virion and is therefore exposed to proteases. *In vitro* cleavage by proteases, such as chymotrypsin, trypsin, and cathepsin L, occur within solvent exposed hypersensitive regions, such as resides 211–215 and 238–244 of serotype 3 virions [101,115]. Importantly, the rates of σ3 cleavage in serotype 3 reovirus (T3D) could be increased by substitution at C-terminal positions 344, 347, and 353, and to a lesser extent by other residues in positions 1–265 [101]. These findings demonstrate the importance of multiple domains on the efficiency of σ3 cleavage.

Several variants of reovirus with distinct mutations in σ3 have been found to exhibit increased rates of uncoating [130,131]. Most well studied is a T3D reovirus variant selected from persistently infected L929 cell cultures containing a mutation in σ3 at position 354 (Y354H). This variant showed increased rates and efficiencies of σ3 degradation and subsequent µ1 to δ cleavage during *in vitro* proteolysis. The Y454H variant was capable of infecting cells treated with ammonium chloride or pan-cysteine protease inhibitor E64, suggesting it is capable of uncoating in the absence of lysosomal proteases [130,132,133,134]. The phenotype of Y354H was reversed by a secondary glycine-to-aspartate change at position 198 in close 3-dimensional proximity (Figure 3) [133]. In the context of immunodeficient mice, the Y354H variant replicated and disseminated more proficiently than wild-type reovirus after intramuscular or peroral administration, causing increased lethality [135]. As for application to oncolytic therapy, the increased pathogenesis of Y354H may raise safety concerns in immunosuppressed individuals. However, given the multifaceted specificity of reovirus towards tumors in immunocompetent models and limited toxicity in humans, it is possible that Y354H would exhibit improved infectivity towards cancer cells that express low cathepsin levels or towards tumors that have fewer extracellular tumor proteases. Furthermore, it was found that mice exposed to the Y354H variants had increased levels of cytokines, such as IFNβ, IL-6, and IFNγ, which could have either positive or negative effects on reovirus oncolysis by either increasing anti-tumor immunity or decreasing virus dissemination respectively. It is also noteworthy that σ3 has dsRNA binding capacity important for evading detection of viral RNAs by cellular antiviral proteins such as PKR; the effects of mutations to σ3 that are aimed towards increasing efficiency of uncoating should therefore be evaluated for potential secondary effects on antiviral signaling [136,137]. Ultimately, a side-by-side comparison of Y354H variant to wild-type reovirus among various immunocompetent tumor models would be highly informative for determining whether enhanced uncoating could enhance oncolytic potency without jeopardizing safety.

### 3.3. Mutations in µ1 Modulate Reovirus Uncoating

As anticipated, the rate and efficiency of uncoating is also modulated by sequence and structure of µ1 (Figure 3). Reovirus serotypes 1 *vs.* 3 exhibit different efficiencies of ISVP-to-ISVP* conversion associated with a polymorphisms in µ1, specifically at position 305 [106,109,138]. Sarkar and Danthi (2013) sought to further explore the role of a loop at residues 72–96 of µ1 during reovirus uncoating, since the loop mediates both µ1-µ1 interactions and associations between µ1 and the virion core proteins σ2 and λ1 [139]. Mutations at position 89 from glutamic acid to alanine (E89A) or glutamine (E89Q) enhanced the capacity for transition from ISVP to ISVP*. Surprisingly, this mutation concomitantly rendered virions *less* (rather than more) infectious. These findings stress the importance of orchestrated uncoating, and that merely making virions less stable is not necessarily favorable. The authors then isolated reovirus variants that reverted to wild-type phenotype. Mutations at residues that lie between µ1 trimers (A276V, D371N) or between µ1 monomers (Q456R, P497S, L185S) were capable of restoring wild-type ISVP-to-ISVP* conversion. Relevant to the quest for improved reovirus potency, when the L185S, A276V, D371N, and Q456R reversion mutations were present alone (*i.e.*, in the absence of the E89Q or E89A mutation), plaques were equal or larger than wild-type reovirus. It would be interesting to determine if these mutations promote plaque size and reovirus replication in cancer cells.

### 3.4. Reduced Virion-Associated σ1 Promotes Reovirus Oncolysis

Following membrane penetration, reovirus particles must fully uncoat into core particles to initiate transcription, protein translation, and new virion production. In a search for mutations that promote reovirus oncolysis, Shmulevitz *et al.* (2012) isolated two variants of the P.W.Lee T3D laboratory strain, T3v1 and T3v2, that had 3–4-fold higher infectivity-per-particle than wild-type reovirus toward a panel of cancer cells [140]. Though only a marginal increase, T3v1 and T3v2 showed exponentially compounding infection over daily round of replication in tumorigenic cells. Importantly, both variants maintained their specificity towards transformed cells. In an aggressive immunocompetent murine model of melanoma, both variants significantly increased survival relative to wild-type reovirus. These variants are among the few that advanced to *in vivo* proof-of-concept testing for the potential to improve reovirus oncolysis through mutations that promote virus replication. T3v2 contained a single mutation in the domain of σ1 involved in anchoring σ1 trimers in virions (S18I). The key phenotype-conferring mutation in T3v1 was in the extra-virion flap domain of λ2 (M1101I), which was previously found to transition from closed-to-open conformation during ISVP*-to-core formation and release of σ1 [107,108,141,142,143,144,145,146]. T3v1 also had mutations in the RNA-dependent RNA polymerase λ3 and the inner core protein λ1, though the relevance of these mutations remains to be tested. The improved oncolytic activity of T3v1 and T3v2 was attributed to decreased levels of σ1 cell attachment proteins on incoming virions, and consequently more-efficient production of reovirus cores with un-occluded λ2 vertex, higher yields of viral RNA, and increased onset of productive infection [147]. In further support for the benefit of reduced σ1 on reovirions, increased infectivity could also be attained by reducing σ1 levels on T3wt using RNA interference during virion production. It should be noted that previous analysis by Larson *et al.* (1994) found only a modest difference in pfu/virion with decreasing virion σ1 levels, and concluded that σ1 levels do not influence infectivity [148]. A discrepancy between the Shmulevitz *et al.* (2012) and Larson *et al.* (1994) findings might reflect differences in the laboratory strain used or differences in assay sensitivities and interpretations. These studies suggest that in the context of the P.W.Lee T3D laboratory strain, 3–5 σ1 trimers are optimal for reovirus infectivity towards tumor cells.

## 4. Macromolecular Synthesis, Progeny Virus Production and Virus Release

Upon accessing the cytoplasm, the conformationally-rearranged cores become transcriptionally active.

Packaged within the cores are the reovirus RNA-dependent-RNA polymerase λ3 and co-factor µ2 which mediate synthesis of viral RNAs. Viral RNAs are then capped as they exit through the pentameric λ2 channels at each core vertex. Reovirus proteins are then synthesized using the host translation machinery. *De novo* reovirus proteins and positive-sense RNAs assemble into progeny core particles. Double-stranded RNAs are then synthesized within progeny cores and serves as a template for more mRNAs that exponentially amplify reovirus replication. Virion replication and assembly occurs within localized centers called virus factories. Several proteins including structural protein µ2 and non-structural proteins µNS and σNS facilitate virus factory formation. Ultimately, reovirus cores are completely assembled by addition of outer capsid proteins µ1 and σ3, and are released from the cell. The time to complete one life cycle of reovirus can vary among cells, but reovirus generally reaches peak titers and release between 18–24 h post-infection of tumorigenic L929, Ras-transformed NIH-3T3 mouse fibroblasts and permissive human cancer cells. This means that a mere four-fold enhancement of any stage of reovirus infection could produce up to 16,000× more infectious virions through exponential replication and cell-cell spread in a single week.

With regards to mutations that promote these steps, a proline at position 208 of reovirus µ2, which is already present in serotype 3 but not in serotype 1 reoviruses, is associated with distinct virus factory morphology and improved virus replication. However, there has, remarkably, been no additional mutations, to our knowledge, that promote post-entry steps of T3 virus replication in permissive cells. It is unclear whether this reflects a lack of research that focuses on promoting post-entry replication steps, or that post-uncoating processes are already optimal in permissive cancer cells. If steps of reovirus replication and release are sub-optimal in the human-imposed tumor niche, then increasing the efficiency of post-entry replication will likely promote yield, dissemination and oncolytic potency of reovirus.

## 5. Improving Reovirus Specificity and Safety

### 5.1. Modifying Reovirus-JAM-A Binding

The safety of using reovirus during cancer therapy was originally supported by the established non-pathogenic nature of reovirus during natural infections. Nevertheless, both dose and route of administration differs considerable when reovirus is used as a cancer therapy relative to normal environmental virus exposure. Over the past decade, numerous clinical trials have therefore tested the safety of reovirus in clinical trials, and have demonstrated minimal side-effects in reovirus-treated patients. Maximizing therapeutic virus safety will however always remain a worthwhile pursuit.

While exploring the fate of cancer cells that become persistently infected by reovirus, Kim *et al.* (2007) discovered a reovirus variant that exhibits reduced toxicity in mice models [149,150]. Human HT1080 fibrosarcoma cells exposed to reovirus develop clonal populations of virus-resistant cells. Interestingly, reovirus-resistant cells were found to be persistently infected and importantly, to have lost their tumorigenic potential *in vivo*. The attenuated reovirus (AV-Reo) extracted from HT1080 virus-resistant cells had several mutations in the viral σ1 cell attachment protein and σ3 outer-capsid protein (Table 1). Most notably, a pre-mature stop codon resulted in truncation of σ1 after the sialic-acid binding domain (Figure 2). Given the loss of the JAM-A binding domain in σ1, the authors sought to evaluate the oncolytic activity and specificity of AV relative to wild-type reovirus. *In vitro*, AV-Reo showed similar infectivity and virus protein synthesis relative to T3wt among HT1080 cells, tumorigenic mouse L929 fibroblasts, and HCT116 human colorectal carcinoma cells. However, AV-Reo did exhibit reduced cytopathic effect (CPE) in L929 and HCT116 cells, suggesting that this variant is somewhat attenuated. When injected intra-tumorally into xenografts established from human colorectal HCT116 cells or HT1080 cells, both AV- and wild-type reovirus caused similar and almost-complete reduction in tumor volumes. Remarkably, AV-Reo showed fewer side-effects *in vivo* relative to wild-type reovirus. For example, AV-reovirus was less cytopathogenic towards normal embryonic stem cells (ESCs) and *in vivo* teratomas established from ESCs. Whereas wild-type reovirus caused myocarditis in severe combined immunodeficiency (SCID) mice, immunohistochemical analysis found limited staining for AV-reovirus in hearts and no signs of heart tissue damage by hematoxylin and eosin staining. Reovirus-associated black-tail syndrome and animal morbidity was also reduced in AV-reovirus-treated SCID mice. Interestingly, two other reovirus variants that were isolated from distinct persistently-infected cultures of Raji or CA46 cells also had mutations in reovirus σ1 and σ3 proteins, but retained a full-length σ1 cell attachment protein and also exhibited toxicity towards immunosuppressed mice. Together, these studies suggest that reovirus containing σ1 devoid of the JAM-A-binding head domain may provide continued anti-tumor activity with improved safety in immunosuppressed patients.

The finding that AV-reovirus showed reduced toxicity in immunocompromised mice is congruent with studies that demonstrated a requirement for JAM-A during hematogenous dissemination of reovirus [151]. Specifically, a strain of serotype 3 reovirus deficient in sialic acid binding [152] (to eliminate confounding effects by sialic acid) was introduced perorally into wild-type (WT) or JAM-A-null newborn C57B mice. Titers of reovirus in the intestine and enteric spread among littermates was similar in JAM-A^−/−^ and WT. This finding suggests a JAM-A- and sialic acid-independent mode of entry is possible during enteric infections. Nevertheless, JAM-A was required for dissemination of reovirus to the liver, heart and brain, and for clinical signs of disease and morbidity. The lack of JAM-A toxicity by AV-Reo, may therefore represent a phenotype similar to JAM-A deficient mice. An important question that arises from these studies is whether JAM-A binding is required for dissemination of reovirus to metastatic sites. It would be prudent to determine if AV-Reo can efficiently reach a tumor when injected intravenously, and spread to sites of metastasis. If JAM-A binding is required for spread of reovirus to sites of metastasis, then the benefits of reduced toxicity would have to be carefully weighed against the oncolytic benefits. Alternatively, the JAM-A binding head of σ1 could be engineered to bind novel tumor-specific target molecules, as discussed elsewhere in this review.

### 5.2. Increasing Interferon Sensitivity

While normal healthy cells are well equipped to detect virus pathogen-associated molecular patterns (PAMPS), produce cytokines such as interferons, and upregulate antiviral proteins that thwart infection, tumor cells often acquire deficiencies in these pathways. Though the mechanisms for reduced antiviral signalling in cancer cells is poorly understood, it is thought to arise from the pressure on tumors to evade immune detection. Like most viruses, reovirus possesses strategies to hamper the antiviral response. For example, while most RNA viruses expose dsRNA replication intermediates in the cytoplasmic that can be detected by PAMP-receptors (e.g., RIG-I, MDA5), reovirus maintains its dsRNA within the core particle at all times. Reovirus transcripts synthesized by viral core particles are capped at the 5' end by enzymatic activities of viral λ2 and co-factor µ2, which avoids recognition of uncapped 5' triphosphate RNA by RIG-I. The σ3 protein of reovirus binds and sequesters viral RNAs from PAMP-receptor detection [136,153,154,155,156]. Finally, reovirus µ2 alters subcellular localization of interferon regulatory factor 9 (IRF9) needed for expression of interferon-stimulated genes (ISGs) [157,158]. Using reassortant analysis, Sherry *et al.* (1998) also found a role for viral core proteins σ2 and λ2 in sensitivity to IFN-β [159]. Nevertheless, reovirus remains some-what sensitive to IFNs and ISGs, and differential activation of antiviral signaling is one of several mechanisms for specificity of reovirus towards tumor cells [29,32,33].

Specificity of some oncolytic viruses towards cancers has been improved by inactivating their antiviral evasion mechanism. For example, the γ34.5 gene product of herpesvirus-1 (HSV-1) usurps cellular PP1α phosphatase to inactivate TBK1 (a kinase involved in antiviral signaling) and to restore activity of eIF2α required for continued protein translation [160,161,162,163]. Removal of γ34.5 diminishes neurovirulence of HSV-1 and contributes to oncolytic specificity. The inability of non-human-tropic viruses such as Newcastle disease virus (NDV), vesicular stomatitis virus (VSV), and parvovirus minute virus of mice (MVM) to overcome antiviral response in human cells also contributes to their specificity towards tumors [164,165,166]. With respect to reovirus, it would be interesting to determine if further debilitation of antiviral evasion strategies would promote tumor-specificity and safety.

Using chemical mutagenesis, Rudd and Lemay (2005) selected reovirus variants with increased sensitivity to interferon and therefore increased specificity towards IFN-deficient transformed cells [33]. Specifically, variants P4L-12 showed ~1000-fold reduced titers in interferon-treated L929 cells relative to wild-type reovirus. In normal NIH3T3 mouse fibroblasts, which are highly proficient at antiviral signalling, there was minimal viral protein expression by P4L-12. In contrast, P4L-12 replicated robustly in Ras-transformed NIH3T3 fibroblasts that have deficient IFN signaling [32]. Genome sequencing of P4L-12 identified mutations in several reovirus genes (Table 1) [75]. To discover which mutation corresponds with increased IFN sensitivity, Sandekian and Lemay (2015) generated recombinant viruses by site-directed mutagenesis and reverse genetics. A threonine-to-methionine change at amino acid position 636 of λ2 was sufficient to confer IFN sensitivity. This residue lies in one of two methyltransferase domains of λ2, and may therefore contribute to the efficiency of mRNA capping and recognition by cellular proteins that bind 5' triphosphate RNAs (e.g., RIG-I). Direct evidence for differences in mRNA capping would strengthen this proposed mechanism. Nevertheless, these studies provide proof-of-concept for the possibility of increasing sensitivity of reovirus to antiviral responses and thereby promoting specificity towards IFN-deficient cells. Based on this premise, it is likely also possible that modifications to reovirus σ3 and µ2 could enhance specificity of reovirus by increasing IFN-sensitivity. For example, a proline at position 208 of µ2 was found to be important for repression of IFN signaling [157]. Basic residues in the carboxyl terminus of σ3, such as R236, R239, K291, and K293, were found to be necessary for dsRNA binding [136,167]. It was also demonstrated that interactions between the amino-terminus of σ3 and the outer capsid µ1 protein, which are necessary for reovirus assembly, alter the subcellular distribution and dsRNA binding activity of σ3 [167,168]. A temperature sensitive mutant of reovirus (ts453) with an N16K mutation in σ3 that diminishes µ1 association shows improved interferon resistance [169]. These domains of µ2 and σ3 could potentially be modified to decrease their protective effects against IFN and PKR activation, thereby making reovirus more specific for IFN-deficient cells.

While the rationale for increasing reovirus specificity by modulating virus attachment or IFN sensitivity is logically sound based on *in vitro* studies, it is critical that these concepts be evaluated *in vivo*. Since oncolytic activity of reovirus is mediated through both direct virus-killing and indirect promotion of anti-tumor immunity, a comparison of wild-type reovirus-to-reovirus variants that increase antiviral signaling or sensitivity is pivotal in immune-competent cancer models. Such studies would indicate the contribution of IFN activation and sensitivity to anti-tumor responses. Furthermore, while Ras-transformed NIH3T3 cells exhibit undetectable IFN signaling, these cells provide the extreme-case-scenario. Cancer cells differ widely in the extent of their ability to produce or respond to IFNs. Accordingly, it is also important that IFN-sensitive reovirus variants be tested in a panel of human cancer cell xenografts in immune deficient mice, to ensure that reovirus proliferation and potency of direct-killing is not jeopardized.

## 6. Reovirus Reverse Engineering and Expression of Exogenous Genes

Reverse engineering systems for dsRNA viruses such as reovirus lagged behind other RNA and DNA viruses but were finally achieved by several progressive efforts. In 1999, recoating genetics was used to reconstitute wild-type reovirus cores with engineered forms of outer-capsid proteins *in vitro* [141,170]. In the early 2000s, plasmid-guided *in vitro* transcribed RNAs were successfully incorporated into infectious reovirions following transfection-infection methods [171,172,173,174,175]. This system demonstrated the feasibility of reverse genetics but was impeded by the need to separate input from recombinant virus. In 2007 came the first completely plasmid-based system for reovirus reverse genetics [176,177,178,179]. Each of 10 reovirus genome segments were cloned upstream of a bacteriophage T7 RNA promoter to preserve authentic RNA 5' ends. The antigenomic hepatitis delta virus ribozyme was used to generate authentic RNA 3' ends. Evolution of the system from using vaccinia-expressed T7 RNA polymerase, to a T7 RNA polymerase-expressing baby hamster kidney (BHK) cell line, to plasmid-derived T7 polymerase led to increased efficiency of the system; as did convergence of multiple reovirus genome segments into a 4-plasmid system. The reverse genetics plasmids were kindly deposited into the Addgene plasmid repository and are now widely in use. Reovirus reverse genetics now permits rationale design of reovirus variants with improved oncolytic activity, and importantly, simultaneous incorporation of multiple advantageous modifications within single next-generation oncolytic reovirus platforms.

**Figure 4:**
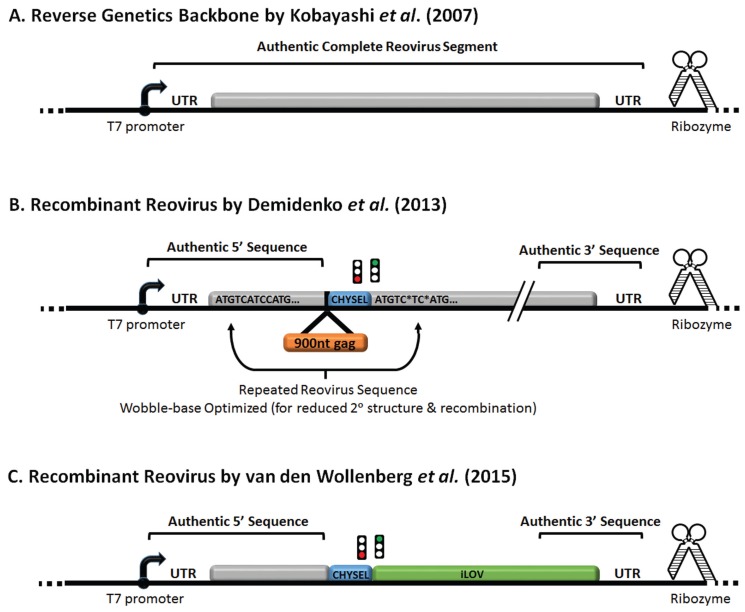
Recombinant reovirus containing exogenous sequences. Illustration of reovirus reverse genetics constructs containing T7 RNA polymerase promoter and ribozyme flanking the 5' and 3' UTR respectively. Reovirus gene segment: (**A**) unmodified, (**B**) encoding SIV-gag, or (**C**) encoding iLOV, were generated as described in the text. In (B) wobble base mutations are denoted by asterisk (*). Constructs were cloned into vector backbone denoted by ellipsis (…).

The ability to express exogenous proteins from reovirus has advanced alongside the reverse genetics systems and most recently has become possible. First, reovirus S2 and S4 genes were replaced by either chloramphenicol transferase or GFP, respectively [171,177]. Replication of these recombinant reoviruses required trans-expression of the authentic reovirus proteins σ2 or σ3, making these vectors less practical as oncolytic therapies. Demidenko *et al.* (2013) generated reovirus vectors that express over 300 amino acids of exogenous protein sequence [180]. Their vectors focused on inserting exogenous polypeptides into either the 3' or 5' ends of reovirus segments S3, M1, or L1 (Figure 4). In-frame tandem duplication of 3' or 5' sequences ensured maintenance of cis-acting signals for RNA packaging, transcription and translation. To prevent recombination and reversion to wild-type sequences, wobble-positions of the inner repeat were mutagenized while conserving amino acid sequence. The 2A-like CHYSEL (*cis*-acting hydrolase element) element from Thosea asigna virus was used to express a both the exogenous peptide and authentic reovirus protein via ribosome skipping. Interestingly, reduction of RNA secondary structure by wobble-mutagenesis was important for increasing the size-allowance of inserts. Using this approach, recombinant reoviruses expressing simian immunodeficiency virus Gag polypeptides were generated. Gag-expressing reovirus could illicit a strong antigen-specific splenic CD8^+^ IFNγ^+^ T-cell response in mice. In the context of oncolysis, similar strategies could be applied to introduce exogenous genes that increase the oncolytic potency of reovirus. For oncolytic purposes, however, it would be important to ensure that exogenous gene expression does not impinge unfavorably on the efficiency of reovirus replication in tumors.

As mentioned above, it was previously demonstrated that truncated σ1 with retained sialic acid binding capacity but no JAM-A binding head domain is sufficient for reovirus infectivity in some cancer cells and potentially a safer alternative due to reduced toxicity towards healthy stem cells [92,149]. van den Wollenberg *et al.* therefore replaced the ~25 kDa head domain of σ1 with a porcine teschovirus-1 2A-like CHYSEL element followed by the small green fluorescent protein iLOV [181]. Recombinant iLOV-Reovirus caused equal-or-more infectivity and cell killing in JAM-A-deficient cells. Future single- and multi-step growth curve analysis in a variety of cancer cells, in addition comparisons in an *in vivo* tumor model, will aid in appreciating the relative fitness of wild-type *vs.* iLOV-reovirus. The authors also indicate that loss of iLOV was seen during repeated virus propagation and rapid-quenching of the iLOV fluorophore; hurdles that will undoubtedly be overcome. In the future, additional small binding motifs could also be added to enhance virus binding affinity and specificity beyond sialic acid recognition.

Several groups have also successfully modified reovirus proteins to alter surface epitopes or binding specificity. The C-terminus of the S1-encoding σ1 cell attachment protein was found to tolerate a hexahistidine tag or up to three consecutive HA tags (Figure 2 depicts C-terminus of σ1) [98,182]. Cell binding and replication of these tagged-σ1 virions was moderately impaired and will benefit from future optimization. As previously mentioned, addition of the hexahistidine tag permitted binding to JAM-A-deficient cancer cells that were modified to express a single-chain tag-specific antibody and demonstrate the potential for further engineering of reovirus receptor specificity in the future. Using the *in vitro* recoating genetics approach, Rouault and Lemay (2003) also incorporated an epitope tag at the C-terminus of the S4-encoding σ3 (Figure 3 depicts C-terminus of σ3) [183]. Though modification of σ3 decreased virus uncoating efficiency, this strategy could be optimized using plasmid-based reverse genetics to introduce better-tolerated modifications to σ3.

The rapid progression of reovirus reverse engineering opens a plethora of possibilities to promote the potency of reovirus oncolysis. Strategies could seek to reduce neutralization by pre-existing antibodies, improve virus binding to JAM-A deficient cancers, optimize the stability-instability balance towards tumor microenvironments, express tumor antigens or cytokines that promote tumor immune responses, or promote bystander tumor cell killing. Such strategies are already in-play for other oncolytic viruses, such as VSV, vaccinia virus, and adenovirus, which are more-easily modifiable and have increased protein coding potential.

## 7. Future Directions

Over the last decade, studies have focused on demonstrating that reovirus holds promise as an oncolytic therapy. With overwhelming evidence that reovirus does exhibit some efficacy and is safe, and with recent advancement of oncolytic viruses towards clinical use, it is now time to focus on bringing reovirus to its ultimate oncolytic potential.

Copious modifications await to be discovered and applied to improve the specificity, safety and potency of oncolytic reovirus. For decades, reovirus provided a safe and powerful tool for mechanistic studies in virology, leading to an abundance of basic research on reovirus structure and replication. Basic discoveries can now help guide well-informed innovations to the reovirus oncolytic vector. One strategy to improve oncolytic reovirus potency would be to improve virus replication and dissemination in the tumors. Given that reovirus evolved for a specialized niche in nature, we believe that adaptation to the human-imposed tumor niche will yield important advancement in oncolytic potency. Improved reovirus infectivity towards tumors would not only increase direct cell-killing, but presumably would increase the indirect benefits of promoting anti-tumor immunity. While this review summarizes many ideas that might improve reovirus replication in tumor cells, it is evident that only few of these ideas have progressed to testing in *bona fide* tumor models. It should also be noted that genetic and phenotypic differences between laboratory strains of T3D are widely accepted and commonly used as tools to understand reovirus evolution and gene function. More direct head-to-head comparisons of modified reovirus variants *in vivo*, especially in the same genetic background, will helps us understand the implication of specific modifications in the context of tumors, metastatic sites, and the immune system.

With the advent of efficient reverse genetics approaches, and novel strategies to add exogenous sequences to reovirus, modifications could strive to promote additive features important for reovirus oncolysis. For example, advancements could aim to enhance systemic delivery of reovirus to tumors and metastatic sites. A recent body of literature suggests that blood cells might assist in transport of reovirus to tumors [184,185,186]. Replication-competent reovirus could be recovered from peripheral blood mononuclear cells, granulocytes, and platelets following intravenous administration in human patients. Once the molecular mechanisms of cell carriage are unravelled, modifications to reovirus that promote association with specific immune cells could promote this process. To test the importance of systemic reovirus clearance by pre-existing and post-treatment neutralizing antibodies, variants of reovirus could be created with changes in immunodominant epitopes and administered in successive cycles of treatment.

The therapeutic value of reovirus, like other oncolytic viruses, also depends on stimulation of anti-tumor immunity for tumor clearance and hopefully reduced reoccurrence [187,188,189,190,191,192,193,194,195,196]. Accordingly, advancements to reovirus should also strive to promote anti-tumor immunity. For example, the ability of cancer therapies to promote immunogenic cell death (ICD), where immune-stimulating danger-associated molecular patterns (PAMPs) are exposed by dying tumor cells, is emerging as an important hallmark of therapeutic potency [197,198,199,200,201,202,203,204,205]. While some oncolytic viruses are suggested to induce ICD, this is not an intuitive consequence of viral infections since viruses tend to evolve strategies to dodge immune detection. It is therefore important that we learn whether reovirus induces immunogenic cell death in tumors, and if modifications could enhance reovirus’ immunotherapeutic value. Finally, additional measures of safety and potency could be introduced into reovirus, such as riboswitches [206,207], prodrug converting enzymes or suicide genes (e.g., ganciclovir-sensitizing HSV thymidine kinase), or miRNA-sensitive sequences. Advantageous modifications could then be combined into united platforms that are most-potent and most-safe, thereby exploiting the true multimodal capacity of viruses. Concurrent advancements in combination-therapy strategies and antitumor immune stimulation will provide a most-optimal regimen and hopefully, a sustained response.
